# Genetic Biomarkers of Metabolic Detoxification for Personalized Lifestyle Medicine

**DOI:** 10.3390/nu14040768

**Published:** 2022-02-11

**Authors:** Lucia Aronica, Jose M. Ordovas, Andrey Volkov, Joseph J. Lamb, Peter Michael Stone, Deanna Minich, Michelle Leary, Monique Class, Dina Metti, Ilona A. Larson, Nikhat Contractor, Brent Eck, Jeffrey S. Bland

**Affiliations:** 1 Department of Nutrition Science, Metagenics, Inc., Aliso Viejo, CA 92656, USA; ilonalarson@metagenics.com (I.A.L.); brenteck@metagenics.com (B.E.); 2 Stanford Prevention Research Center, Department of Medicine, Stanford University, California, CA 94305, USA; 3 Nutrition and Genomics Laboratory, Jean Mayer USDA Human Nutrition Center on Aging, Tufts University, Boston, MA 02111, USA; jose.ordovas@tufts.edu; 4 Nutritional Genomics and Epigenomics Group, IMDEA-Food, 28049 Madrid, Spain; 5 University Camilo José Cela, Villafranca del Castillo, 28692 Madrid, Spain; 6 Bennet Data Sciences, San Diego, CA 92107, USA; andrey@bennettdatascience.com; 7 Personalized Lifestyle Medicine Center, Gig Harbor, WA 98332, USA; josephlamb@plmc.com (J.J.L.); mstone@ashlandmd.com (P.M.S.); dinametti@plmc.com (D.M.); 8 Institute for Functional Medicine Federal Way, Washington, DC 98003, USA; deannaminich@hotmail.com (D.M.); monique@thecffm.com (M.C.); 9 Ashland Comprehensive Family Medicine-Stone Medical, Ashland, OR 97520, USA; 10 Office of Personalized Health and Well-Being, Medical College of Georgia, AU/UGA Medical Partnership, Athens, GA 30606, USA; 11 Human Nutrition and Functional Medicine, University of Western States, Portland, OR 97213, USA; 12 Vida Integrated Health, Seattle, WA 98112, USA; mleary@thinkvida.com; 13 The Center for Functional Medicine, Stamford, CT 06905, USA; 14 Nutrilite Health Institute, Buena Park, CA 90621, USA; nikhat.contractor@amway.com; 15 Personalized Lifestyle Medicine Institute, Bainbridge Island, WA 98110, USA; jeffbland@plminstitute.org

**Keywords:** detoxification, biotransformation, biomarkers, nutrigenomics, single nucleotide polymorphisms, personalized lifestyle medicine, pragmatic clinical trials, functional medicine, environmental health, LIFEHOUSE study

## Abstract

Metabolic detoxification (detox)—or biotransformation—is a physiological function that removes toxic substances from our body. Genetic variability and dietary factors may affect the function of detox enzymes, thus impacting the body’s sensitivity to toxic substances of endogenous and exogenous origin. From a genetic perspective, most of the current knowledge relies on observational studies in humans or experimental models in vivo and in vitro, with very limited proof of causality and clinical value. This review provides health practitioners with a list of single nucleotide polymorphisms (SNPs) located within genes involved in Phase I and Phase II detoxification reactions, for which evidence of clinical utility does exist. We have selected these SNPs based on their association with interindividual variability of detox metabolism in response to certain nutrients in the context of human clinical trials. In order to facilitate clinical interpretation and usage of these SNPs, we provide, for each of them, a strength of evidence score based on recent guidelines for genotype-based dietary advice. We also present the association of these SNPs with functional biomarkers of detox metabolism in a pragmatic clinical trial, the LIFEHOUSE study.

## 1. Introduction

Every day, we are exposed to hundreds of toxic substances of endogenous and exogenous origin, including radical oxygen species produced during cellular respiration and foreign compounds known as xenobiotics such as environmental toxicants, food additives, and drugs. Detoxification(detox) and biotransformation are physiological functions that removes these substances from our body in three separate phases (I to III). Defective detox due to genetic factors, environmental overload, and nutrient deficiencies can result in various chronic diseases, including cancer, asthma, obesity, cardiovascular disease, diabetes [1], and neurodegenerative conditions such as Alzheimer’s disease [2].

Genetic variants and dietary factors may affect the function of Phase I and Phase II detox, thus impacting the body’s sensitivity and response to toxic exposures. In Phase I, functionalization enzymes break down xenobiotics in the liver, producing highly reactive free radical molecules as a byproduct. These activated xenobiotics can damage cell structure and function unless further processed in Phase II or neutralized by endogenous or exogenous antioxidants. In Phase II, conjugation enzymes join activated xenobiotics with large molecules to produce water-soluble substances that are finally excreted from the body in Phase III, the elimination phase, mainly via urine or stools. Several common single nucleotide polymorphisms (SNPs, minor allele frequency ≥1%) have been reported to affect the function of Phase I and Phase II detox enzymes and the manner dietary factors modulate these enzymes. However, the scientific validity and clinical utility of these SNPs are still unclear as most of the current knowledge relies on observational studies in humans or experimental models in vivo and in vitro, with very limited proof of causality and clinical value.

This study provides health practitioners with a review and analysis of state-of-the-art research on genetic variants that could be used for personalized metabolic detoxification programs. In the first section of the paper, we provide a list of SNPs located within genes involved in Phase I and Phase II detoxification reactions, for which some evidence of clinical utility exists. In the second section of the paper, we present the association of these SNPs with functional biomarkers of detox metabolism in the Lifestyle Intervention and Functional Evaluation—Health Outcome Survey (LIFEHOUSE), a real-world pragmatic clinical trial (PCT).

## 2. Clinically Tested Genetic Variants within Genes Involved in Phase I/Phase II Detox Reactions

Table 1 provides a list of clinically tested single nucleotide polymorphisms (SNPs) located within genes involved in Phase I and Phase II detoxification reactions. A list of foods and nutrients interacting with those SNPs to modulate detox metabolism is presented in Table 2. We have selected these SNPs based on their association with inter-individual variability of detox metabolism in response to certain nutrients in the context of human clinical trials. For each of these SNPs, we provide a strength of evidence score based on recent guidelines for the interpretation and classification of nutrigenetic variants [3]. All SNP alleles are reported on the forward strand as in the dbSNP database and some direct-to-consumer genetic reports (e.g., 23andMe).

### 2.1. Phase I Detox Enzymes

The cytochrome P450 (CYP450) superfamily of enzymes constitutes oxidase enzymes that use oxygen to metabolize toxic compounds of exogenous or endogenous origin (e.g., pharmaceuticals, environmental toxicants, and some metabolites of steroid hormones) in Phase I detoxification reactions and synthesize various molecules within cells such as cholesterol, steroids, and bile acids. Phase I enzymes have the potential to induce oxidative damage due to the production of reactive electrophilic species, which require Phase II detoxification (conjugation) in order to be safely cleared from the body.

#### 2.1.1. Cytochrome P-450 1A2

Cytochrome P-450 1A2 (CYP1A2) is a Phase I detoxification enzyme. CYP1A2 activates polycyclic aromatic hydrocarbons (PAHs) from cigarette smoke, car exhaust, and charbroiled foods to carcinogenic intermediates [4]. Various dietary factors and xenobiotics can induce the expression of the *CYP1A2* gene, including caffeine, cruciferous vegetables, paracetamol, aflatoxin B1, and cigarette smoke [4,5]. The rs762551 variant is an intron variant that has been reported to affect the inducibility of *CYP1A2* expression by xenobiotics such as caffeine or cigarette smoking, likely through effects on gene transcription or splicing [6,7,8]. The rs762551(C) allele has been reported to be a “slow-metabolizing allele,” whereas the rs762551(A) allele is “a fast-metabolizing allele.” C-allele carriers produce an enzyme variant with a 62–70% lower activity and are less inducible by xenobiotics than the enzyme variant produced by those with the AA genotype [9,10,11]. Low CYP1A activity can result in decreased detoxification, lower 2/16-alpha hydroxyestrone ratio, and a higher risk of certain cancers [12,13]. On the other hand, increased activity of CYP1A without an increase in Phase II clearance may result in the accumulation of intermediates that are more reactive than the original toxins, resulting in increased oxidative damage and cancer risk [14,15].

Caffeine is an inducer and substrate of CYP1A2. C-allele carriers are “slow” caffeine metabolizers compared to those with the AA genotype, as evidenced by lower levels of caffeine metabolites such as 1,7-dimethylxanthine and 1,7-dimethyluric after a dose of caffeine [9]. A prospective study of individuals screened for stage 1 hypertension [16] and two case–control studies in myocardial infarction (MI) patients [17,18] suggest that C-allele carriers should limit coffee consumption to less than 1 cup/day or caffeine from other drinks to less than 100 mg/day to avoid being at higher risk of hypertension and myocardial infarction. In contrast, those with the AA genotype are “rapid” caffeine metabolizers and may benefit from consuming 1–4 cups of coffee/day due to increased consumption of phytonutrients presumed to be protective against heart disease.

Cruciferous vegetables (broccoli, Brussels sprouts, cauliflower, watercress, and cabbage) may increase CYP1A2 activity. In contrast, apiaceous vegetables (carrots, celery, dill, parsley, and parsnips) may decrease it, but these effects do not seem to depend on the *CYP1A2* genotype. In a randomized, crossover feeding trial, the consumption of cruciferous vegetables for two weeks increased CYP1A2 activity in a dose-dependent manner in all rs762551 genotypes compared with a low-phytochemical diet, with men experiencing greater dose–response than women [19]. In the same study, the consumption of apiaceous vegetables and cruciferous vegetables for two weeks decreased *CYP1A2* activity compared with a diet containing cruciferous vegetables alone [19].

#### 2.1.2. Cytochrome P450 1B1

Cytochrome P450 1B1 (CYP1B1) is a Phase I detoxification enzyme that localizes to the endoplasmic reticulum (ER) and metabolizes procarcinogens and estrogens. For example, it converts 17beta-estradiol to 4-hydroxyestradiol, a mutagenic metabolite that has been implicated in breast and endometrial carcinogenesis.

The rs1056836(G) allele of the CYP1B1 gene changes an amino acid in the protein sequence from valine to leucine, which has been associated with lower enzyme activity [20]. This may result in reduced activation of toxicants, reduced production of 4-hydroxyestrogens, and reduced oxidative damage. As a result, individuals with GG and CG genotypes may have greater protections against exposure to pro-carcinogens than those with the CC genotype [21]. In contrast, individuals with CC genotypes tend to have higher enzymatic activity and produce 2–4 times more 4-hydroxyestrogen than those with the GG genotype [22]. They might, hence, be at higher risk for estrogen-associated cancers. However, the effects of this SNP on cancer risk are not clear and may depend on age, ethnicity, and menopausal status [23,24].

The consumption of fruit juice rich in the flavonoid quercetin, a modulator of CYP450 activity [25], has been reported to reduce oxidative stress to a greater extent in rs1056836(G)-allele carriers than in those with the CC genotype. In a clinical trial in 168 healthy volunteers, the consumption of a blueberry/apple juice providing 97 mg quercetin and 16 mg of ascorbic acid a day for 4-weeks reduced ex vivo H_2_O_2_-provoked oxidative DNA damage to a greater extent in lymphocytes from G-allele carriers than in those with the CC genotype [20].

### 2.2. Phase II Detox Enzymes

#### 2.2.1. Glutathione S-Transferases Mu 1 (GSTM1) and Theta 1 (GSTT1)

Glutathione S-transferases mu 1 (GSTM1) and theta 1 (GSTT1) are Phase II enzymes involved in detoxifying harmful electrophilic compounds of endogenous or exogenous origin, including environmental toxicants, therapeutic drugs, and products of oxidative stress such as lipid peroxidation products. This is accomplished by the conjugation of glutathione to highly reactive Phase I intermediate metabolites to produce water-soluble substances that can be removed from the body. Radical oxygen species and a variety of foods, phytonutrients, and xenobiotics can induce the production of these enzymes, possibly by the upregulation of the Nrf2 signaling pathway.

Many people carry genetic deletions in one or both copies of *GSTT1* and *GSTM1* genes. These deletions have different frequencies in different ethnic groups, being present in more than 70% of Caucasians and Asians but less than 25% of Africans [26,27]. Deletions of both copies of the *GSTM1* or *GSTT1* genes are often referred to as double deletions. Individuals carrying *GSTM1* or *GSTT1* double deletions may have a decreased ability to detoxify environmental toxicants, carcinogens, and products of oxidative stress. This results in an increased risk of developing cancers [28] but also in increased sensitivity to chemotherapy and better treatment outcomes [29,30,31]. The effect of GST genotype on chemotherapy sensitivity highly depends on the type of drug used (e.g., alkylating agents or kinase inhibitors).

*GSTM1* and *GSTT1* deletions can modulate the effects of certain nutrients on GST activity and the required intake of those nutrients for adequate support of detox reactions. Individuals carrying gene deletions in *GSTM1* or *GSTT1*, especially those carrying deletions in both genes, may have a more rapid excretion of bioactive nutrients found in cruciferous vegetables such as isothiocyanates and sulforaphane [32,33]. In particular, individuals with *GSTM1* null/null genotype (double deletions) were found to excrete ~30% more urinary sulforaphane and other isothiocyanates than *GSTM1*-positive individuals in both a randomized crossover trial in 16 healthy adults [32] and a feeding study in 114 healthy adults [33]. Consequently, GST double deletion carriers may need to consume more crucifers than those who carry at least one copy of *GSTM1* or *GSTT1* to experience health benefits. This may explain why observational studies report greater cancer-protecting benefits for double deletion carriers vs. GST-positive individuals only in European and Asian populations [34,35,36] but not in the United States [37,38,39,40], probably due to lower intakes of cruciferous vegetables in the US compared to Europe and Asia.

Clinical studies report that cruciferous vegetables increase the activity of GST enzymes to a greater extent in those who carry a *GSTM1* or *GSTT1* deletion compared to GST-positive individuals [22,41]. However, others did not confirm this association [42,43,44]. In line with these findings, GST double deletion carriers show a greater increase in GST-mediated detoxification than GST-positive individuals after consuming cruciferous-based supplements. In a randomized crossover trial in 82 smokers, oral 2-phenethyl isothiocyanate (PEITC), a compound from the watercress, which is a cruciferous plant, increased the urinary excretion metabolites of carcinogens found in cigarette smoke (i.e., mercapturic acids of benzene and acrolein) [45]. Smokers with *GSTM1* null/null genotype experienced higher increases in mercapturic acids of benzene (43%) compared to the placebo controls than the individuals with one or two gene copies (11%). If they also had a double deletion of the GSTT1 gene, they experienced even greater increases in urinary mercapturic acids of benzene (95.4%) after PEITC supplementation. In contrast, those who had at least one copy of both *GSTM1* and *GSTT1* did not experience any detox-enhancing effect from PEITC supplementation.

However, gender and lifestyle factors such as smoking can modify the interaction between GST genotype and dietary factors on GST activity. Cruciferous vegetables may increase GST activity in those who carry deletions of both copies of *GSTM1*, with effects more pronounced in females than in males [22,41,45]. In contrast, apiaceous vegetables seem to inhibit *GSTM1* specifically in men, not women, who carry at least one copy of the *GSTM1* gene. These effects were observed in a clinical study of adult non-smokers after consuming either cruciferous vegetables or apiaceous vegetables for a period of 6-days [22].

Antioxidants-rich food may decrease oxidative stress to a greater extent in GST double deletion carriers than GST-positive individuals. In a clinical trial referenced above, individuals with two copies of the *GSTT1* gene benefited more from the consumption of a blueberry/apple juice providing 97 mg quercetin and 16 mg ascorbic acid a day with respect to reducing risks of oxidative DNA damage compared with individuals with one copy or with double gene deletions [20].

Smokers who carry GST deletions may particularly benefit from supplementation with antioxidants because carcinogens in cigarette smoke can overload their detox capacity and induce a higher production of ROS byproducts. However, antioxidants seem to improve certain oxidative stress markers such as glutathione levels and vitamin C to a greater extent in those with at least one copy of *GSTM1* or *GSTT1*. In a study in 95 adult Korean male smokers, all participants experienced significant reductions in DNA damage upon supplementation with purple grape juice. The antioxidant impact of the grape juice differed based on the genotype. Only individuals with one or two copies of the *GSTT1* gene (*GSTT1* present/present or present/null) experienced significant increases in blood vitamin C concentrations. In addition, only individuals with double *GSTM1* deletions (null/null genotype) experienced significant increases in plasma vitamin E concentrations. In contrast, glutathione levels were increased in those with one or two copies of the *GSTM1* gene (*GSTM1* present/present or present/null) [46].

#### 2.2.2. Catechol-O-Methyltransferase (COMT)

Catechol-O-methyltransferase (COMT) is involved in phase II (conjugative) metabolism of various molecules possessing catechol structure, including catecholamines (such as dopamine, epinephrine, and norepinephrine), estrogens, drugs (e.g., L-DOPA), and xenobiotics including tea catechins and benzo[a]pyrene metabolites (found in tobacco smoke, grilled meats, and other foods). In this process, COMT transfers a methyl group from S-adenosyl methionine (SAM) to a catechol compound, thereby producing S-adenosyl homocysteine (SAH), a homocysteine precursor. This reaction is essential for the detoxification of catechol compounds and the maintenance of appropriate dopamine and norepinephrine levels for optimal brain health (e.g., behavior, cognition, and stress management).

The rs4680 SNP in the *COMT* gene determines a G to A substitution (forward DNA strand), which in turn produces a substitution from valine (Val) to methionine (Met) at position 158 of the amino acid sequence of the enzyme (Val158Met). This substitution affects COMT’s activity, with the variant A (Met) allele producing an enzyme with 40 % lower activity than that encoded by the ancestral G (Val) allele [47,48]. This may result in decreased degradation of neurotransmitters (dopamine, epinephrine, and norepinephrine), estrogen, drugs, and other catechol compounds. As a result, A-allele carriers may have increased sensitivity to environmental toxicants, a higher risk of developing neuropsychiatric disorders, and impaired estrogen metabolism. For example, individuals with genotype AA may have a higher risk of developing breast cancer upon exposure to endocrine-disrupting chemicals (EDCs) [49].

The rs4680 SNP may affect the metabolism of certain nutrients and their effects on *COMT*-mediated detox reactions. Tea catechins such as epigallocatechin-3-gallate (EGCG) are a *COMT* substrate and may increase *COMT* activity shortly after consumption when consumed at high doses (500–1000 mg) [50]. Individuals with the AA genotype, who have slow *COMT* activity, may be slow catechin metabolizers, have a lower catechin excretion in the urine, and retain more catechins in the blood than those with the GG genotype [51,52]. As a result, they may benefit from a lower intake of tea catechins. In contrast, those with the GG genotype, who have higher *COMT* activity, may be more sensitive to the short-term effects of tea catechins, such as an increase in insulin secretion and blood pressure (BP). These effects were observed in three randomized clinical trials that used high doses of green tea or green tea extract (GTE) consumed before a high carbohydrate meal [53,54,55].

Individuals with the GG genotype, who have higher COMT activity, may experience the health benefits of olive oil and red wine at lower intakes than those required for individuals with the AA genotype. This is due to a greater ability to convert hydroxytyrosol, a phenolic compound in virgin olive oil and red wine, into its cardioprotective metabolite homovanillyl alcohol (HVAL). In a prospective cohort study in 1851 elderly adults at high risk of cardiovascular disease (CVD), individuals with the GG genotype had significantly higher urinary concentrations of HVAL compared to those with the AA genotype [56].

#### 2.2.3. Bilirubin Uridine Diphosphate Glucuronosyl Transferase

Bilirubin uridine diphosphate glucuronosyl transferase (bilirubin-UGT) is a Phase II detoxification enzyme produced by the *UGT1A1* gene that conjugates glucuronic acid to a variety of endogenous and exogenous substances such as bilirubin, estrogen, dietary carcinogens, and several medications to facilitate their excretion from the body. This reaction plays an important role in converting toxic unconjugated bilirubin to non-toxic conjugated bilirubin and is involved in the metabolism of 40–70% of all drugs.

The rs3064744(TA) allele, also known as UGT1A1*28 and previously annotated as rs34815109 or rs34983651, is an insertion variant resulting from the addition of an extra TA nucleotide pair in the promoter region of the *UGT1A1* gene. While the -/- and -/TA genotypes do not seem to have any functional or clinical impact, the homozygous (TA/TA) genotype reduces the amount of the bilirubin-UGT enzyme produced by transcription, resulting in a 70% decrease in enzymatic activity [57]. Consequently, TA/TA individuals may be at higher risk for certain cancers [58] and experience increased toxicity in response to certain drugs such as the chemotherapeutic irinotecan and the antiallergenic drug tranilast [59,60,61]. Some TA/TA individuals may develop Gilbert syndrome, a benign condition characterized by increased serum levels of total and unconjugated bilirubin and associated with a lower risk of coronary artery disease (CAD). Not all TA/TA individuals develop Gilbert syndrome, indicating the involvement of environmental factors and other genetic variants, such as those in *UGT1A6* and *UGT1A7* genes, that further regulate the glucuronidation process.

Cruciferous vegetables and apiaceous vegetables may decrease total serum bilirubin levels in rs3064744(TA) allele carriers, with greater effects observed for TA/TA homozygous subjects. In a randomized, controlled, and crossover feeding trial, 70 healthy nonsmoking adults with genotype TA/TA and TA/- experienced, respectively, a 16–21% and 8% decrease in bilirubin, a marker of *UGT1A1* activity, upon daily consumption of a single or double dose of cruciferous vegetables or a single dose of cruciferous vegetables plus a single dose of apiaceous vegetables for two weeks [42]. Similarly, citrus fruit may help lower serum bilirubin in rs3064744(TA) allele carriers, although these effects may be limited to women with the TA/TA genotype. In an observational study in healthy nonsmoking adults, women with the TA/TA genotype who consumed ≥0.5 daily servings of citrus fruit had ~30% lower serum bilirubin than those with the same genotype who consumed less, whereas -/- and TA/- genotypes did not experience any effects [62].

## 3. Validation of Genetic Variants of Detox Metabolism in Real-World Clinical Settings

We next explored the association of the genetic variants presented herein with functional biomarkers of detox metabolism within the Lifestyle Intervention and Functional Evaluation—Health Outcome Survey (LIFEHOUSE), a real-world pragmatic clinical trial (PCT) conducted at the Personalized Lifestyle Medicine Center (PLMC) by Metagenics Inc. The study design and protocol have been described in detail previously [63]. Briefly, LIFEHOUSE utilizes an “N-of-1” and a Tent-Umbrella-Bucket design to define functional markers of health and to model interventions facilitating the adaptation of personalized lifestyle medicine recommendations in an employee health population. For genotyping, version 5 of the 23andMe chip was used.

Gene deletions in GSTM1 and GSTT1 as well as TA insertions in *UGTA1* were not included in our analysis as the SNPs used to assess these genetic variants or their surrogates were not available on version 5 of the 23andMe chip. As functional biomarkers of detox metabolism, we evaluated the blood levels of gamma-glutamyltransferase (GGT), homocysteine, and oxidized low-density lipoprotein (oxLDL), as these biomarkers have been identified as reflecting complex interactions between environmental toxicity and physiological disturbances underlying common diseases of aging. Lee and Jacobs have noted that serum GGT levels within its reference range are a sensitive marker of oxidative stress and may provide a valuable marker of exposure to persistent organic pollutants identified as a risk factor for the development of Type 2 Diabetes Mellitus [64,65]. Homocysteine has been demonstrated to be a risk factor for cardiovascular and neurodegenerative disorders with suspected mechanisms including disordered mitochondrial energy production, nitric oxide and peroxynitrate signaling, and oxidative stress [66,67,68]. Oxidized LDL, reflecting a key precipitating step in endothelial dysfunction and atherosclerosis, may represent a global marker of oxidative stress [69,70].

**Figure 1:**
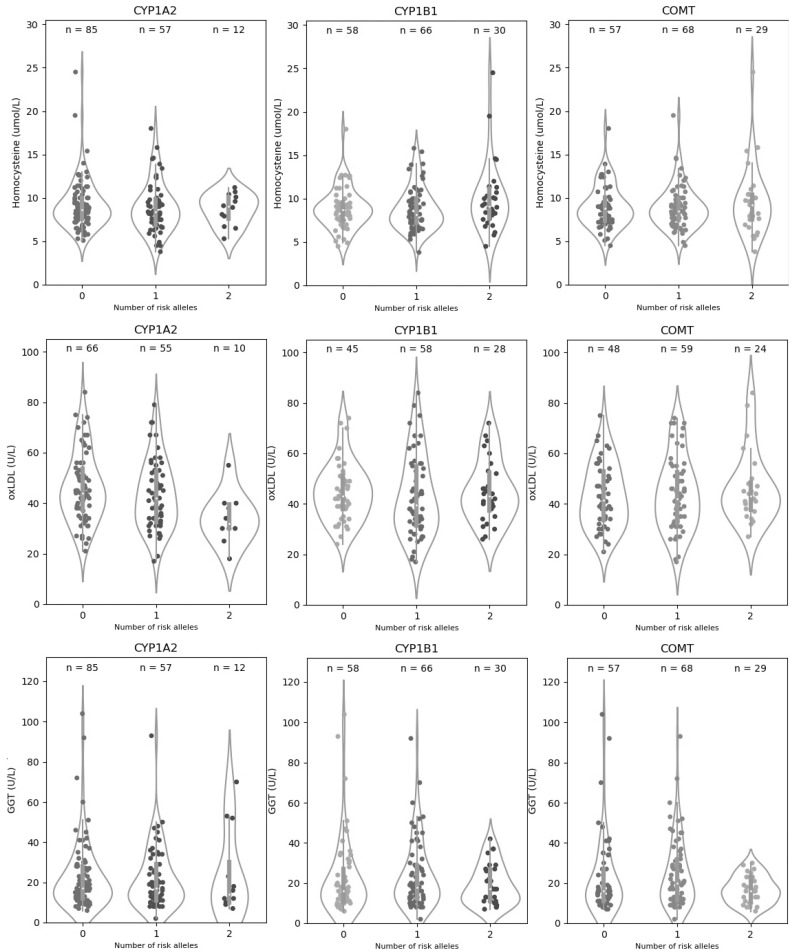
Distribution of homocysteine, oxLDL, and GGT values by genotype. The violin plots show the density plot and statistical summary of homocysteine, oxLDL, and GGT values for each genotype of the CYP1A1, CYP1B2, and *COMT* genes. The white dot of the box depicts the median, the thick gray bar in the center represents the interquartile range (IQR), which indicates the spread of the middle half of the distribution, and the thin line extending from the gray bar represents the rest of the distribution lying between the ±1.5× IQR range. The points represent the actual distribution of individual data. On each side of the gray line is a smoothed estimation of probabilities for new points, calculated using the Kernel Density Estimation, showing the distribution shape of the data. Wider sections of the violin plot represent a higher probability that members of the population will have the given value; the skinnier sections represent a lower probability.

Table 3 and Table 4 show, respectively, the baseline levels of GGT, homocysteine, and oxLDL in the study population and their association with the investigated genetic variants. As shown in Table 4, carrying the effect allele of the genetic variants in *CYP1A2* (rs762551-C), *CYP1B1* (rs1056836-C), or *COMT* (rs4680-A) correlated with worse detox function or increased oxidative damage, as indicated by higher levels of homocysteine, oxLDL, or GGT. Violins plots in Figure 1 present a visualization of the distribution and statistical summary of the data. Heterozygous subjects tended to have intermediate biomarker levels between those carrying two copies of the effect allele and those carrying two copies of the protective allele. Notably, the alleles linked to lower activity of Phase I enzymes (*CYP1A2* rs762551-C, and *CYP1B1* rs1056836-G) were associated with greater toxic load in the liver, as indicated by elevated GGT, but lower oxidative damage, as indicated by lower levels of oxLDL and/or homocysteine. In particular, rs762551-C in *CYP1A2* was associated with substantially and significantly lower oxLDL. This likely reflects the importance of balancing Phase I and Phase II reactions for effective and safe detoxification as the products of Phase I detox reactions are highly reactive. These metabolites can produce increased oxidative stress unless promptly cleared by Phase II enzymes. Among all associations, only the one between *CYP1A2* rs762551 and oxLDL was statistically significant (*p* ≤ 0.05). It is important to note, however, that due to the small sample size, our study was underpowered with respect to detecting potentially clinically significant differences in detox outcomes. Therefore, any statistically significant association or lack thereof must be interpreted with caution.

Next, we calculated a polygenic risk score (PRS), including all the above SNPs, to assess their cumulative impact on detox function. In order to calculate PRS, we assigned one point for each effect allele carried by an individual (e.g., zero, one, and two copies) and summed the points for all genetic variants in the score. An equal weight was assigned to each genetic variant. Subjects were ranked by PRS and divided into tertiles corresponding to high, intermediate, or low genetic risk. Genetic risk, as predicted by PRS, did not display a stronger association with homocysteine, oxLDL, or GGT than compared to single SNPs. One of the reasons for this finding is that the “risk allele” as defined in the medical literature does not always correspond to a low-activity protein. This results in discrepant biomarker associations across different variants that, when combined in a PRS, may show worse prediction power than single variants used alone. For example, the risk allele of *CYP1A2* is the one that produces a lower activity protein, whereas the risk allele of *CYP1B1* produces a high activity protein. Therefore, the risk alleles of *CYP1A2* and *CYP1B1* show opposite associations with homocysteine, oxLDL, and GGT, and combining these variants into one PRS reduces the power to detect abnormal values in these biomarkers. This suggests that, when dealing with ambivalent risk alleles such as *CYP1A2*, clinicians may need to use personalized PRSs in which the risk allele is ad hoc chosen based on their patients’ individual biomarker imbalances and health goals.

## 4. Conclusions

Thus far, only six variants in genes encoding Phase I and Phase II detox enzymes have been tested in clinical studies: *CYP1A2* rs762551; *CYP1B1* rs1056836; segmental deletions of GSTT1 and GSTM1; *COMT* rs4680-A; and insertion variants of *UGTA1* (TA/-). None of these variants has been consistently associated with detox-related outcomes in the context of human clinical trials. Therefore, clinicians should always use them in conjunction with functional testing to pinpoint patients that might benefit from specific detox interventions. We identified interesting correlations between SNPs and functional biomarkers of detox function and oxidative stress in our real-world clinical, but still pilot, setting. An important a limitation of this analysis was the lack of information on participants’ diet and health status to correlate with genotype and markers of oxidative stress and functional detox. Combining these SNPs in a polygenic risk score (PRS) may enhance their prediction power. However, clinicians must use caution when dealing with genetic variants that can be regarded as risk factors or protective factors depending on an individual’s characteristics and health goals. Future studies should validate these variants and their combination in PRSs in large, well-designed clinical studies that investigate the effects of specific interventions on detox-related outcomes and use common standards to define dietary protocols and stratify patients to evaluate heterogeneity of response in different patient subgroups.

## Figures and Tables

**Table 1:** Common genetic variants within genes involved in Phase I/Phase II detox reactions associated with variability of response to foods or nutrients that modulate detox metabolism.

Phase I Detox Enzymes		
*CYP1A2*		
Effect allele	Allele frequency	Effects on enzymatic function
rs762551-C Strength of evidence: Convincing (A).	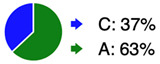	C-allele carriers produce an enzyme variant with 62–70% lower activity and are less inducible by xenobiotics. Low CYP1A activity can result in decreased clearance of toxins, a lower 2/16-alpha hydroxyestrone ratio, and a higher risk of certain cancers. Consequently, lower production of reactive detoxification intermediates may reduce oxidative stress.
*CYP1B1*		
Effect allele	Allele frequency	Effects on enzymatic function
rs1056836-C Strength of evidence: Possible (C).	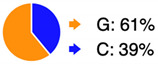	Individuals with the CC genotype tend to have higher enzymatic activity than G-allele carriers, which may result in greater activation of toxicants, greater production of 4-hydroxy estrogens, and greater oxidative damage. The effects of this SNP are affected by age, ethnicity, and menopausal status.
**Phase II detox enzymes**		
*GSTM1*		
Effect allele	Allele frequency	Effects on enzymatic function
*GSTM1* deletion *GSTT1* deletion Strength of evidence: Probable (B).	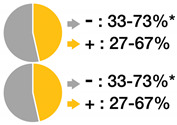 * -: deletion; +: present	Individuals carrying *GSTM1* or *GSTT1* double deletions (-/- genotype) may have a decreased ability to detoxify environmental toxicants, carcinogens, and products of oxidative stress. Gene deletions are more frequent among Caucasian and Asian populations and less frequent in African populations. Different segmental deletions have different frequencies in the population and between different ethnicities.
*COMT*		
Effect allele	Allele frequency	Effects on enzymatic function
rs4680-A Strength of evidence: Probable (B).	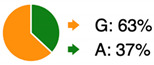	The A allele (Met) produces an enzyme with 40 % lower activity than that encoded by the G allele (Val). A-allele carriers may have a decreased ability to degrade neurotransmitters, estrogen, and various xenobiotics. This may result in increased sensitivity to environmental toxicants, a higher risk of developing neuropsychiatric disorders, and impaired estrogen metabolism.
*UGT1A1*		
Effect allele	Allele frequency	Effects on enzymatic function
rs3064744-TA Strength of evidence: Possible (C).	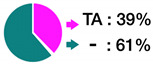	Individuals carrying two insertion alleles (TA/TA genotype) may have a lower enzymatic activity than those carrying at most one copy of the deletion allele (-). This may result in increased toxicity in response to certain drugs (acetaminophen) and to a benign cardio-protective condition known as Gilbert syndrome, characterized by increased serum levels of total and unconjugated bilirubin.

Abbreviations: SNP identification numbers (noted as “rs...”) are the unique SNP identifiers from the NCBI dbSNP database. *CYP1A2*: Cytochrome P-450 1A2; *CYP1B1*: Cytochrome P-450 1B1; *GSTM1*: Glutathione S-transferase mu 1; *GSTT1*: Glutathione S-transferase theta 1; *COMT*: Catechol-O-methyltransferase; *UGT1A1*: UDP-glucuronosyltransferase A-1.

**Table 2:** Foods and nutrients that modulate the activity of Phase I/Phase II enzymes and their interaction with genotype.

Food/Nutrient	Gene	Effects on Enzymatic Function
Caffeine	*CYP1A2*	Caffeine is an inducer and substrate of *CYP1A2*. rs762551-C allele carriers are “slow” caffeine metabolizers, and they should limit coffee consumption to <1 cup/day or caffeine from other drinks to <100 mg/day to avoid being at higher risks of hypertension and myocardial infarction. In contrast, those with the AA genotype are “rapid” caffeine metabolizers and may benefit from consuming 1–4 cups of coffee/day due to increased consumption of phytonutrients presumed to be protective against heart disease.
Cruciferous vegetables (broccoli, Brussels sprouts, cauliflower, watercress, and cabbage)	*CYP1A2*	May increase *CYP1A2* activity, but it is unclear whether the magnitude of this effect may depend on CYP1A genotype.
	*GSTM1*, *GSTT1*	Individuals carrying gene deletions in *GSTM1* or *GSTT1*, especially those carrying deletions in both genes, may have a more rapid excretion of bioactive nutrients found in cruciferous vegetables such as isothiocyanates and sulforaphane. Consequently, they may need to consume greater amounts of cruciferous vegetables than those who carry at least one copy of either *GSTM1* or *GSTT1*. On the other side, double-deletion carriers tend to experience a greater increase in GST activity and GST-mediated detoxification upon consumption of cruciferous vegetables or cruciferous-based supplements such as 2-phenethyl isothiocyanate (PEITC). The GST-inducing effects of cruciferous vegetables are more pronounced in females than in males.
	*UGT1A1*	May decrease serum bilirubin levels in rs3064744-TA allele carriers with greater effects observed for TA/TA homozygous.
Apiaceous vegetables (carrots, celery, dill, parsley, parsnips, etc.)	*CYP1A2*	May decrease *CYP1A2* activity, but it is unclear whether the magnitude of this effect may depend on CYP1A genotype. May exert inhibitory effects on GSTM-1 in men, not women, who carry at least one copy of the *GSTM1* gene.
	*GSTM1*, *GSTT1*	May exert inhibitory effects on GSTM1 in men, not women, who carry at least one copy of the *GSTM1* gene.
	*UGTA1*	May decrease serum bilirubin levels in rs3064744-TA allele carriers with greater effects observed for TA/TA homozygous.
Quercetin and antioxidant rich foods (citrus fruits, apples, onions, red wine, olive oil, dark berries, etc.)	*CYP1B1*	Quercetin may reduce oxidative stress to a greater extent in rs1056836-G allele carriers than in those with the CC genotype. These findings were made with quercetin from fruit juices at doses significantly lower (~100 mg) than those typically used for supplementation (500–1000 mg).
	*GSTM1*, *GSTT1*	Quercetin and other antioxidants from blueberry, apples, and purple grapes may reduce oxidative stress to a greater extent in GST double deletion carriers than GST-positive individuals. Smokers who carry GST deletions may especially benefit from supplementation with antioxidants because carcinogens in cigarette smoke can overload their detox capacity and induce a higher production of ROS byproducts. However, quercetin and other antioxidants seem to improve certain oxidative stress markers such as glutathione levels and vitamin C to a greater extent in those with at least one copy of GSTM-1 or GSTT-1.
Tea catechins	*COMT*	Individuals with the rs4680 AA genotype, who have slow *COMT* activity, may be slow catechin metabolizers and retain more catechins in the blood than those with the GG genotype. As a result, they may benefit from a lower intake of tea catechins. In contrast, those with the GG genotype, who have higher *COMT* activity, may be more sensitive to the short-term effects of tea catechins, such as an increase in insulin secretion and blood pressure (BP).
Olive oil, red wine	*COMT*	Individuals with the rs4680 GG genotype, who have higher *COMT* activity, may experience the health benefits of olive oil and red wine at lower intakes than those with the AA genotype. This is due to a greater ability to convert hydroxytyrosol, a phenolic compound in virgin olive oil and red wine, into its cardioprotective metabolite homovanillyl alcohol (HVAL).
Citrus fruit	*UGT1A1*	May help lower serum bilirubin in women with the rs3064744 TA/TA genotype. These effects may be noticeable in all TA allele carriers.

Abbreviations: SNP identification numbers (noted as “rs...”) are the unique SNP identifiers from the NCBI dbSNP database. *CYP1A2*: Cytochrome P-450 1A2; *CYP1B1*: Cytochrome P-450 1B1; GST: Glutathione S-transferase; *GSTM1*: Glutathione S-transferase mu 1; *GSTT1*: Glutathione S-transferase theta 1; *COMT*: Catechol-O-methyltransferase; *UGT1A1*: UDP-glucuronosyltransferase A-1; ROS: Radical Oxygen Species.

**Table 3:** Baseline demographics and functional biomarkers.

	Mean (SD)
	*n* = 157
Age, years	43 (11)
Sex	
Female	106 (68%)
Male	51 (32%)
Ethnicity	
Caucasian	77 (49%)
Asian	20 (13%)
African American	4 (2%)
Mediterranean	5 (3%)
Northern European	14 (9%)
Native American	6 (4%)
Other	19 (12%)
Homocysteine (µmol/L)	9.1 (3)
Missing	3 (2%)
oxLDL (U/L)	44 (13.8)
Missing	26 (16%)
GGT (U/L)	22.5 (16.7)
Missing	3 (2%)

**Table 4:** Association between genetic variants in Phase I/Phase II detox enzymes and levels of homocysteine, oxLDL, and GGT. For each biomarker (rows), we report the mean levels and standard deviation in groups of subjects carrying the same genotype of the selected genetic variants.

	CYP1A2|rs762551-C				CYP1B1|rs1056836-C			
	Genotype	Subjects (%)	Mean (SD)	*p*-Value	Genotype	Subjects (%)	Mean (SD)	*p*-Value
Hcy	AA	85 (55.2)	9.21 (2.89)	0.796	GG	58 (37.7)	8.87 (2.34)	0.232
	AC	57 (37.0)	8.91 (2.90)		CG	66 (42.9)	8.77 (2.46)	
	CC	12 (7.8)	8.71 (1.85)		CC	30 (19.5)	10.05 (4.02)	
oxLDL	AA	66 (50.4)	46.21 (13.80)	0.018	GG	45 (34.4)	44.82 (11.52)	0.459
	AC	55 (42.0)	43.23 (13.75)		CG	58 (44.3)	43.01 (16.12)	
	CC	10 (7.6)	34.00 (10.08)		CC	28 (21.4)	44.85 (12.29)	
GGT	AA	85 (55.2)	22.58 (17.08)	0.862	GG	58 (37.7)	22.67 (19.29)	0.555
	AC	57 (37.0)	21.86 (15.43)		CG	66 (42.9)	23.77 (16.82)	
	CC	12 (7.8)	24.33 (21.39)		CC	30 (19.5)	19.1 (9.95)	
	**COMT|rs4680-A**				**Polygenic Risk Score**			
	**Genotype**	**Subjects (%)**	**Mean (SD)**	***p*** **-Value**	**Genetic Risk**	**Subjects (%)**	**Mean (SD)**	***p*** **-Value**
Hcy	GG	57 (37.0)	9.02 (2.49)	0.853	Low	88 (57.14)	8.79 (2.15)	0.215
	AG	68 (44.2)	8.88 (2.46)		Medium	45 (29.22)	8.96 (2.84)	
	AA	29 (18.8)	9.54 (4.02)		High	21 (13.64)	10.40 (4.53)	
oxLDL	GG	48 (31.2)	43.08 (12.81)	0.728	Low	71 (54.20)	45.92 (13.32)	0.103
	AG	59 (38.3)	43.85 (14.59)		Medium	40 (30.53)	40.45 (13.72)	
	AA	24 (15.6)	46.38 (14.16)		High	20 (15.27)	44.50 (15.07)	
GGT	GG	57 (37.0)	22.93 (19.36)	0.23	Low	88 (57.14)	24.00 (19.25)	0.890
	AG	68 (44.2)	24.44 (17.04)		Medium	45 (29.22)	20.16 (12.15)	
	AA	29 (18.8)	16.83 (6.82)		High	21 (13.64)	20.86 (13.57)	

Abbreviations: Hcy: homocysteine; oxLDL: oxidized low-density lipoprotein; GGT: gamma-glutamyltransferase; PRS: Polygenic Risk Score.

## Data Availability

Data are contained within the article.
